# Not All Remote Workers Are Similar: Technology Acceptance, Remote Work Beliefs, and Wellbeing of Remote Workers during the Second Wave of the COVID-19 Pandemic

**DOI:** 10.3390/ijerph182212095

**Published:** 2021-11-18

**Authors:** Simone Donati, Gianluca Viola, Ferdinando Toscano, Salvatore Zappalà

**Affiliations:** 1 Department of Psychology, University of Bologna, 47521 Cesena, Italy; simone.donati@unibo.it (S.D.); gianlucaviol@gmail.com (G.V.); ferdinando.toscano@unibo.it (F.T.); 2 Department of Psychology and Human Capital Development, Financial University under the Government of the Russian Federation, 125993 Moscow, Russia

**Keywords:** remote working, Technology Acceptance Model (TAM), Work from Home (WFH), well-being, coping

## Abstract

Although a large part of the world’s workforce engaged in mandatory Work from Home during the COVID-19 pandemic, the experience was not the same for everyone. This study explores whether different groups of employees, based on their work and organizational characteristics (i.e., organizational size, number of days per week working from home, working in team) and personal characteristics (i.e., remote work experience, having children at home), express different beliefs about working remotely, acceptance of the technology necessary to Work from Home, and well-being. A study was conducted with 163 Italian workers who answered an online questionnaire from November 2020 to January 2021. A cluster analysis revealed that work, organizational, and personal variables distinguish five different types of workers. ANOVA statistics showed that remote workers from big companies who worked remotely several days a week, had experience (because they worked remotely before the national lockdowns), and worked in a team, had more positive beliefs about working remotely, higher technology acceptance, and better coping strategies, compared to the other groups of workers. Practical implications to support institutional and organizational decision-makers and HR managers to promote remote work and employee well-being are presented.

## 1. Introduction

To contain and proactively react to the spread of the COVID-19 virus, many countries not only implemented social distancing and face mask requirements [1], but they also encouraged organizations to massively adopt remote work practices [2]. This latter action involved an acceleration of trends that were already underway, such as the digitalization of organizational processes [3] and the legal definition and experience remote work [4,5].

In Italy, the percentage of remote workers dramatically increased from 8% of the total workforce in 2019 to about one third of the entire national workforce [6].

The massive adoption of Work from Home (WFH) during the COVID-19 pandemic motivated scholars to investigate the effects of this way of working on employees’ job performance and job satisfaction [7,8,9] and their physical and mental well-being [7,8,10,11,12]. Several studies focused on employees’ experiences during the first lockdown (which in Italy took place from March to May/June 2020). Currently, however, there is a lack of studies about the way people experienced the second lockdown (i.e., from October 2020 to January 2021) and how different types of employees perceived the extended WFH conditions. In this regard, Sanchez-Gomez et al. [13] highlight that Coronavirus pandemic was appraised by workers as a stressful event hindering their mental health. Nevertheless, the role played by WFH on workers’ well-being during the second lockdown still remains under-investigated.

This study aims to identify whether different groups of employees who engage in remote work have different beliefs about the Work from Home experience during the COVID-19 pandemic. To reach this goal, we used the Technology Acceptance Model (TAM) as the primary theoretical model.

The TAM dimensions of perceived ease of use and perceived usefulness have demonstrated great power in explaining users’ intentions to adopt a specific technology or continue to use it [14]; therefore, we used these two dimensions to explore workers’ WFH perceptions. Furthermore, we investigated workers’ beliefs about issues related to remote work [5,15,16] such as work-family balance, relationships with co-workers, and skills needed to use the information technologies required to work remotely. Finally, based on Zito et al. [17] and Kniffin et al. [18], we examined how lockdown conditions and mandatory remote work were related to employees’ well-being. In particular, we proposed that changes in the workplace would be related to employees’ perceptions of their coping capacity and work self-efficacy and the extent to which organizations supported them during the lockdowns.

Based on the literature, we argue that teleworkers’ perceptions and well-being are related to the plans adopted by organizations in implementing the remote work programs [19,20,21], the extent of their adoption (i.e., Hoornweg et al. [22]), the isolation caused by remote work (i.e., Galanti et al. [10]; Toscano & Zappalà [9]), and work-family interferences, especially considering that family duties interfered with work because of closure of schools and child-care services during the lockdowns (i.e., Cuerdo-Vilches et al. [23]).

Scholars developed a large part of the previous literature on remote work before the COVID-19 pandemic. Therefore, it is important to understand whether and how workplace conditions related to telework are related to WFH beliefs, even during the forced lockdowns and the waves of the pandemic [18].

The present study aims to fill the aforementioned research gaps by adopting a two-stage research approach. In the first stage, we identify groups of workers who share similar working conditions, such as general organizational characteristics (e.g., organizational size) and job and personal characteristics related to telework (e.g., number of days spent working remotely or previous remote work experience). In the second stage, we explore whether different groups of remote workers have different perceptions of remote work’s usefulness and ease, work self-efficacy, perceived organizational efficacy, and ability to cope with work demands during the lockdown period. This study aims to contribute to the theoretical literature by examining whether different types of remote workers perceive remote work differently, and whether beliefs about remote work are differentially related to psychological and social predictors. Practical contributions of the study are related to designing and implementing organizational procedures that may support WFH acceptance and motivation in different types of employees who engage in remote work.

### 1.1. Work from Home (WFH) during the COVID-19 Pandemic

Remote work is usually described as a working arrangement that consists of working away from the office or the usual place of work. Thus, it implies working from different locations, such as work-from-home (WFH), working in satellite offices, or working at customer/client/supplier locations [24,25]. Since the early stages of the COVID-19 pandemic, due to national lockdowns implemented to minimize the virus’s spread, WFH has been the “ordinary” way of working for millions of workers [7,10,11]. Prompted by national governments, organizations have implemented WFH mainly for employees with specific working conditions, such as when working with non-material inputs and/or outputs, using digital technologies and ICTs, or in situations, where interactions with colleagues and coordination by managers can be performed remotely [24]. 

The pandemic has created conditions that have indirectly reinforced and, in some cases, accelerated digitalization, urging individuals and organizations to adopt several technologies to manage more flexible, automated, and interconnected work [26]. Similarly, Work from Home involves adopting digital tools that modify working conditions and, thus, might significantly impact workers’ technology acceptance, motivation, and well-being [26]. 

Although previous literature suggests that remote work programs increase workers’ productivity and satisfaction [27,28], recent studies have shown that new technologies for remote work during COVID-19 have well-being costs for employees, such as an increase of technology-related job demands and technostress [12,29,30], but also in terms of anxiety, depression and sleep quality [31]. This evidence has led to the assumption that it is necessary for not only employees, but also managers, to consider the dark sides of pandemic WFH [32]. Given the need to study WFH perceptions during the COVID-19 pandemic, the controversial impact of remote work on employees’ well-being, and the fact that remote work outcomes may depend on specific working conditions, we propose the following research questions:

During the COVID-19 pandemic, have specific remote work conditions shaped workers’ experience by creating differences in their WFH acceptance, beliefs, well-being, and motivation at work?

To answer this question, the present contribution is divided into two studies. In the first study, we run a cluster analysis to investigate subgroups of employees who share personal, work, and organizational conditions. In a second study, we compare the subgroups, testing whether they also differ on their WFH perceptions and well-being. 

### 1.2. Work and Organizational Conditions and Workers’ Profiles 

Empirical studies show that, during the COVID-19 pandemic, remote workers developed different WFH beliefs and well-being perceptions depending on their personal characteristics (such as gender, age, living with children, etc.) or working conditions (e.g., job contract or work in team) [33,34]. Nonetheless, Ipsen and colleagues [24] highlight that research on remote work needs to overcome a single-factor comparison approach (i.e., gender or age group) by using a multi-factor method. Thus, adopting an exploratory approach, we decided to take into account five dimensions that previous literature has empirically found to be related to remote workers’ technology acceptance and well-being. These five dimensions include personal (having child/children, previous remote work experience), work (working in team, number of days spent working remotely), and organizational characteristics (organizational size). Here we briefly describe these five dimensions.

Organizational size. Organization theory recognizes firm size as a factor strongly correlated with organizational structure complexity, a higher internal division of labor, and a more diffuse use of formal control [35]. Literature reviews have revealed mixed results about the relationship between firm size and telework adoption [20,36]. On the one hand, Sarbu [37,38] showed that telework adoption is more diffuse in SMEs due to their more task participative and people-centered culture; on the other hand, Illegems et al. [39] suggested that telework requires a deeper job redesign, resources, and competencies that only larger organizations possess. Home-based telework, especially during COVID-19, has involved essential and sometimes onerous changes in the work organization and the relationship between employees, supervisors, and firms [19]. Thus, considering the mandatory and widespread implementation of WFH during the lockdowns, we assume that large companies devoted more attention and resources to WFH programs and implemented these types of programs earlier.

Remote Work previous experiences. Mandatory WFH has profoundly changed organizations’ and employees’ working conditions by requiring them to change scheduling, monitoring, and task communication [33]. Many employees have had to learn to balance work schedules and family responsibilities [34]. Moreover, the obligation to self-arrange an effective home-workstation and use new (sometimes unfamiliar) digital technologies is recognized as a perceived disadvantage of remote work during the lockdowns [24]. Nevertheless, pre-COVID-19 studies showed that familiarity with remote work favors employees’ positive beliefs and acceptance of telework, as well as the intrinsic motivation to use it [20,21]. Furthermore, familiarity with remote work also decreases the perceived anxiety about its adoption. Considering the above, it is reasonable to argue that workers with previous remote work experiences (i.e., started working remotely before the COVID-19 lockdowns) have better knowledge and competencies that are useful for managing mandatory WFH. Therefore, we expect workers who are familiar with remote work to develop better WFH acceptance, beliefs, and motivation to use it, compared to workers who did not have past experiences with it.

WFH work days. The amount of time a person works remotely in a given time frame (e.g., one week) has long been considered a factor that modulates the relationship between telework and several outcomes [36]. Previous research found that the effects of the time spent working remotely may be curvilinear: little or a lot of telework seems to lead to the worst results in terms of job satisfaction, whereas about 15 working hours (approximately 2.5 days) a week should be optimal for this relationship [40,41]. Analogous conclusions have been drawn when individual performance was the dependent variable. Hoornweg, Peters, and Van Der Heijden [22] found that maximum productivity was reached at around eight hours of remote work per week, and then productivity decreased as the number of hours working remotely increased. The number of hours (or days) in a week devoted to working remotely also moderates the effect of professional isolation on job performance [42]. Even studies conducted during the pandemic took into account the hours or days of remote work in a week. During the pandemic, for example, hybrid (part at the office and part at home) work formulas were shown to be more effective in maximizing the positive relationship between social support by supervisors and colleagues and employees’ vigor [43], suggesting that, when preferred, telework is an option that should be accompanied by a period of returning to the office. Given the power of the “number of WFH days”, we think it may distinguish and characterize different groups of homeworkers and be differentially related to technology acceptance and well-being.

Having children. Having and taking care of children has always been considered a driver of telework [15]. During the pandemic, the relationship between remote work and family life management was more complex. For many employees, the mandatory nature of home work was associated with the burden of managing children who were also at home because schools and other educational agencies were offering their services remotely. As a result, the houses of home workers during the pandemic were quite crowded, and various studies observed that the presence of children at home was associated with employees’ decreased physical and psychological well-being [34], enhanced perceived workload, especially for women [44], and boundary management issues [45,46]. On the other hand, significant differences in stress and work-family conflict were observed between women with or without children [11]. Therefore, we believe that the presence of children at home should be taken into account when studying the experience of home workers during the pandemic, and we consider it a potential stressor for employees.

Work in team. For employees with individual jobs characterized by limited collaboration with co-workers, mandatory WFH represents fewer opportunities for face-to-face interactions [34]. The physical distance from managers and colleagues forced by lockdowns might increase remote workers’ social isolation [42], and may create tension between colleagues, especially if not all the group members Work from Home [47]. Previous studies have shown that lack of personal contacts and teamwork is related to a decreased perception of the usefulness of telework and managers’ intentions to not adopt it [21,48]. In addition, collaborating with other co-workers engaged in teleworking contributes to developing a supportive “community of practices” in which to exchange information about the job, but also about how to effectively manage WFH activities and stress [48]. It is reasonable that remote workers working in a team have more opportunities to interact remotely with co-workers, receiving and giving social support. For this reason, we expect teleworkers involved in teamwork to have more positive beliefs and greater acceptance of WFH and a higher perception of well-being, compared to employees who perform their job alone.

Based on the above, and adopting an exploratory approach, this study investigates whether different levels of the personal, work, and organizational characteristics just described identifying different groups of employees. In addition, it investigates whether these identified groups of employees also differ on the remote-work-related variables presented in the next section.

### 1.3. Work from Home (WFH), Technology Acceptance Model (TAM), and Beliefs about Remote Work

Literature on the acceptance of technology has become particularly relevant in explaining how individuals’ perceptions of a specific technology affect their intention to use or reject that technology, as well as its actual usage. The Technology Acceptance Model (TAM) [49], one of the most widely used models in this regard, postulates that specific individual beliefs about a specific technology influence users’ intention to adopt and use that technology [14]. Inspired by the theory of Planned Behavior [50], TAM has been used to study a broad range of technologies or technologically innovative solutions, such as email and ICTs [51,52] or more recent digital technologies, such as wearable technologies, Internet of Things or big data [26,53,54], and telework [19,21]. 

Specifically, the first version of the TAM suggests that the perceived ease of use and perceived usefulness of a technology influence the actual use of that technology [55]. Perceived usefulness refers to “the extent to which a person believes that using an Information Technology (IT) will enhance his or her job performance”, whereas perceived ease of use refers to “the degree to which a person believes that using an IT will be free of effort” (Davis et al. [49], p. 275). Based on previous studies that used TAM to investigate remote work [19,20,25], we examined remote workers’ beliefs about WFH, perception of ease of WFH, and perception of WFH’s usefulness. Beliefs allow us to consider cognitive schemas of favorableness toward a specific technology that stem from direct or indirect (through others) experience [25]. Beliefs can guide the encoding of information, attention, and behaviors in the workplace [56], and they represent an important source of influence of employees’ technology-use intentions and their actual usage [25].

In our study, we considered three beliefs about WFH that previous literature reviews [5,15] and empirical surveys [24,57] have highlighted as crucial points in remote workers’ experience: work-family balance, workplace relationships, and ICT technical skills needed to work remotely. 

Regarding work-family balance, working from home makes the boundaries between work and family roles more permeable [15,57]. Thus, WFH requires workers to learn to effectively manage the invasion of the “home world into one’s work time, as well as the intrusion of work tasks into one’s personal life” (Aczel et al. [57], p. 2). Workplace relationships are also impacted by WFH because it means “being disconnected from co-workers, experiencing isolation due to the physical and social distance to the team and office members” (Aczel et al. [57], p. 2). In fact, remote workers at home often do not have the opportunity to socialize and give/obtain social support from colleagues [34]. Finally, working remotely also implies some level of ICT technical skills. Remote work requires organizations to implement digital infrastructures and ICT solutions [19], and teleworkers have to develop familiarity and confidence with the technological infrastructure and software to Work from Home [20] by acquiring or using the specific technical competencies needed to work remotely [58]. We argue that positive experiences with work-family balance, workplace relationships, and the necessary technical skills are indicators of positively oriented beliefs about WFH.

### 1.4. WFH Workers’ Motivation and Well-Being: Worker Self-Efficacy, Coping Strategies, and Organizational Effectiveness

Finally, we argue that being forced to suddenly adapt their life and work practices to anti-COVID-19 protocols, such as the adoption of WFH, and also continuous exposure to COVID-19 news and job-related demands [18], might also affect employees’ psychological work conditions. Xiao and colleagues [34] argued that the extended stay at home during the pandemic may have changed life and work habits, leading to social isolation, depression, and anxiety feelings. The decline in mental health and well-being due to changes made after the appearance of COVID-19 [59] also seems related to physical and psychological stress produced by radical modifications in the work domain, especially for employees who were reluctant to adopt the mandatory WFH solution [18]. Following the job demands—control theory [60], WFH during COVID-19 might represent, especially for employees not previously trained and prepared for remote working, a potentially stressful situation of augmented job demands and decreased control over their job and their perceptions of efficacy in a broad sense. In addition, previous research suggests that self-efficacy and, potentially, by conceptual extension, organizational efficacy and self-regulatory coping strategies, may act as predictors of good telework adaptation and behavior structuring (a concept similar to self-leadership, e.g., proactive work planning) [61].

Self-efficacy, coping strategies, and organizational effectiveness, in other words, may be affected by the work situation generated by the pandemic, but they can also act as a resource to achieve better adaptation to it. In light of this potential dual course of action, we expect that workers who have experienced this period of forced WFH as more stressful may have worse perceptions of personal and organizational efficacy. In addition, we also believe that these perceptions of efficacy are associated with more positive worker beliefs about WFH, especially if employees have been using this work arrangement for a long time and, thus, have had more time to adjust to it.

## 2. Materials and Methods

### 2.1. Sample and Procedure

An online questionnaire was posted on the Qualtrics platform and answered by a sample of 163 Italian workers. To contact homeworkers, the link to the questionnaire was distributed on the Internet and social networks (LinkedIn and Facebook) during the second lockdown in Italy, which lasted from mid-November 2020 to mid-January 2021. Reasons for the study and information on safeguarding anonymity were provided at the beginning of the survey. Respondents were free to fill in the questionnaire, not answer, or leave it at any time. Participants reported an average age of 39.89 years (SD = 10.00; min. 19; max. = 65). Females made up 41.1% of respondents, 44.8% reported having a child/children, and most of the participants had at least a university education level (68.7%). The majority of the respondents worked in for-profit private companies (66.25%), had full-time employment (84%), and did their job in collaboration within a team (87.1%). Regarding the size of the organization, 18.4% of respondents worked in micro-sized companies, 8% in small ones, 16.6% in medium-sized enterprises, and 57.1% in large companies. To reach their workplace, 42.3% of the participants declared that they devoted less than 20 min to commuting, 37.4% usually spent from 20 to 45 min, and 20.2% had to travel for more than 45 min. With regard to WFH, 20.8% declared that they worked face-to-face, whereas 79.2% were working from home. In the case of the latter, 40.8% had started working remotely even before the first national lockdown (March–April 2020), whereas 52.3% started WFH during the first lockdown. Home workers stated that they worked from home an average of 4 days per week (SD = 1.57; Min. = 1 day, Max. = 6 days). Table 1 summarizes the respondents’ characteristics.

### 2.2. Measures

Information about participants’ demographic and professional characteristics, current work situation, and organizational information were gathered using single-item, multiple-choice questions. 

Remote Work previous experiences. Participants answered a question about their experience with remote work by choosing one of the following alternatives: “1: I have never experienced remote work”; “2: I started to work remotely during the COVID-19 first lockdown”; “3: I started to work remotely before the first COVID-19 lockdown”. 

Organizational size. Alternatives to indicate organizational size were the following: 1: “micro-sized company (less than ten employees)”; 2: small company (from 10 to 49 employees); 3: “medium-sized company (from 50 to 249 employees)”; 4: “big-size company (up to 250 employees)”.

WFH work days. Respondents indicated, on average, the number of days they worked remotely in a week by selecting a number from “0: none” to “6: six days per week”.

Work in team. Two alternatives were provided to examine whether respondents mainly performed their work activities alone or usually collaborated with co-workers within a team.

Having a child/children. Participants indicated that they were not parents (“1: No”) or that they were parents of a child/children (“2: Yes”).

Psycho-social variables were measured using the following scales:

WFH Acceptance. Based on the Technology Acceptance Model (TAM), six items measured Perceived Usefulness (PU) of WFH (e.g., “Working remotely speeds up the work”; α = 0.88), and six items measured Perceived Ease of Use (PEOU) (e.g., “Remote work is easy to learn”; α = 0.78). The items on the scale used by Davis in 1989 [49] were adapted to this study. A 5-point Likert scale (from 1: “completely disagree” to 5: “completely agree”) was used. 

Remote work beliefs. Beliefs about remote work were examined using three items measuring specific aspects of telework [15,19]: work-family balance, workplace relationships, and technical skills needed. Specifically, participants expressed their agreement with the following statements: “Work from Home allows me to better reconcile working life with extra-working life”; “Work from Home harms the workplace relational network” (reverse item); “I can Work from Home effectively thanks to my IT competencies and skills” on a 5-point Likert scale (from 1: “completely disagree” to 5: “completely agree”).

Coping—Positive reinterpretation of negative or stressful events. To measure how participants coped with the forced WFH, we used the “Positive reinterpretation and growth” subscale from the Italian adaptation of the Cope test [62] developed by Sica et al. [63]. The 4-item scale measures the strategies adopted by responders to create something good from negative or stressful experiences. Examples of items are “I try to use negative experiences to grow as a person” and “I am looking for something positive in what is happening”. Items were rated on a 4-point scale (from 1: “never” to 4: “always”). Cronbach’s α was 0.73.

Perceived Work Self-Efficacy. Personal work self-efficacy was measured with the 4-item scale by Major and Kozlowski [64], adapted and validated in an Italian sample by Sarchielli and Toderi [65]. It consists of 4 items (α = 0.87) rated on a 5-point Likert scale (from 1: “completely disagree” to 5: “completely agree”). Two examples of items are “Thanks to my past experiences, I am prepared to effectively do this job” and “I can achieve my work goals thanks to the training and experience I’ve gained”. 

Perceived Organizational effectiveness. We measured perceived organizational effectiveness using the 7-item subscale from Caprara [66]. Examples of items are “My company is always able to facilitate the introduction of all technological innovations” and “My company is always able to ensure the well-being of its members”. Respondents rated each item on a 5-point Likert scale (from 1: “never” to 5: “always”). Cronbach’s α was 0.86. 

Control variables. By using single-item, multiple-choice questions, we gathered data about gender (“1: female”, “2: male”), age groups (“1: up to 24 years”; “2: between 25–39 years”; “3: from 40 to 54 years”; “4: 55 years and up”), educational level (“1: middle school or lower”; “2: high school”; “3: graduate”; “4: master”; “5: Ph.D. or other Academic specialization”), respondents’ current type of employment (“1: full-time job”; “2: part-time job”), and the amount of time usually dedicated to commuting to the workplace (“1: up to 20 min”; “2: from 20 min to 45 min”; “3: more than 45 min”).

### 2.3. Data Analysis

All the analyses were conducted using IBM SPSS Statistics, version 26. Respondents’ socio-demographic characteristics are reported in Table 1, whereas means and correlations of the focal variables of the study are shown in Table 2.

Considering the exploratory aim of this study, we performed a two-step cluster analysis to examine the presence of natural groupings of respondents. The clustering procedure maximizes intra-group similarities and between-group differences [67]. Organization size, remote work experience, having a child/children, and collaborating in a team were used as categorical and ordinal clustering variables. The number of WFH days per week was considered an interval variable. ANOVA was used to check whether the identified clusters were different, both for the grouping variables and respondents’ characteristics. Finally, we ran an ANOVA to test cluster differences in respondents’ WFH Acceptance, remote work beliefs, coping strategy, work self-efficacy, and organizational effectiveness.

## 3. Results

### 3.1. Cluster Analysis and Remote Workers’ Profile Descriptions

Five different clusters emerged from the cluster analysis. The cohesion and separation silhouette test revealed an average value of 0.3, indicating sufficient internal cluster cohesion and sufficient separation among clusters [67]. Clusters 1, 3, and 4 include 34 respondents each, Cluster 2 is composed of 21 participants, and Cluster 5 contains 40 respondents. The cluster analysis included all the respondents in the 5-cluster classification. 

Cluster 1 is composed only of workers who work in the office, did not Work from Home on any day, and do not have any direct experience with WFH. All of them declared to work in a team, 50% belong to small companies, and 73.5% have no children. Because of their work conditions, we called this group “*in person workers*”.

Cluster 2 comprises 62% of employees who Work from Home (24% of them since before the first lockdown) an average of 2.52 (SD = 2.34) days per week; 57% of the members of this cluster work in small enterprises, 62% have children, and 100% say they mainly perform their job tasks alone. We called this cluster “*the lone workers*”. 

Cluster 3 is characterized (100%) by remote workers who usually Work from Home 3.44 (SD = 1.5) days per week. Three-quarters (76.5%) of the members of this cluster started to work remotely during the first national lockdown. This group’s respondents belong to small and medium enterprises (62% of them work in a medium-sized company), where 100% work within a team. Due to the type of company where they work and the type of work they do, we called this cluster “*Small and Medium Enterprises (SMEs)’ remote workers*”. 

Cluster 4 comprises 100% of remote workers in big-sized companies who started working from home during the first national lockdown an average of 3.79 (SD = 1.47) days per week. All (100%) of this cluster’s members work in a team, and 65% of them have no children. Considering the company characteristics and the remote working experience, we called this cluster “*big companies’ early-stage remote workers*”. 

Cluster 5 includes only remote workers in big-sized companies who perform their jobs within a team and started the remote work experience long before the first national lockdown. Members of this cluster usually Work from Home 3.73 (SD = 1.72) days per week, and 55% of them have a child/children. We called this cluster “*big companies’ experienced remote workers*”. 

Table 3 describes and summaries the distribution of the respondents’ socio-demographic characteristics across the five clusters.

We conducted two ANOVAs to verify that the five clusters differed on both the scores on the grouping variables and the characteristics of the cluster members. Table 4 shows significant differences among the clusters in previous remote work experience (*F*(4, 158) = 159.72; *p* < 0.01), organizational size (*F*(4, 158) = 47.50; *p* < 0.01), and number of WFH days per week (*F*(4, 158) = 37.00; *p* < 0.05). In particular, the Bonferroni Post-hoc test showed that Cluster 5 (*big companies’ experienced remote workers*) had significantly more experience with remote work than the other four groups. Similarly, Clusters 4 and 3 (*big companies’ early-stage remote workers* and *SMEs’ remote workers*) have more extensive experience than the *lone workers* and the *in person workers* clusters. In relation to the average number of WFH days per week, the latter two groups showed lower levels compared to Clusters 3, 4, and 5. Regarding the organizational dimension, Clusters 4 and 5 (*big companies’ early-stage remote workers* and *big companies’ experienced remote workers*) showed significantly higher values than the other three clusters. 

Considering respondents’ characteristics (see Table 5), the *lone workers* show a significantly lower education level than the *big companies’ early-stage remote workers* and *SMEs’ remote workers* clusters. The latter group also has a significantly higher educational level than the *in person workers* group members. In relation to the type of employment and the time spent commuting, Cluster 1 (*in person workers*) has more part-time employment and less commuting time than Cluster 5 (*big companies’ experienced remote workers*).

### 3.2. Remote Workers’ Cluster Profile Comparisons

Table 6 reports the means and standard deviations of the five clusters on the TAM dimensions (PU and PEOU) and the other psychosocial variables, and the ANOVA values.

In the case of the TAM dimensions, the clusters’ mean scores are significantly different, both for PU (*F*(4, 158) = 4.55; *p* < 0.01) and for PEOU (*F*(4, 158) = 6.29; *p* < 0.01). Specifically, Bonferroni post-hoc tests showed that members of Cluster 5 (*big companies’ experienced remote workers*) perceive greater usefulness of WFH than the other four clusters, and they have a higher perception of WFH ease of use compared to Cluster 1 (*in person workers*). Significant differences among the clusters are also observed for beliefs related to work-family balance (*F*(4, 158) = 3.39; *p* < 0.05) and ICT technical skills needed to work effectively from home (*F*(4, 158) = 3.33; *p* < 0.05). Bonferroni post-hoc tests showed that members of Cluster 5 have more positive beliefs about the role played by WFH in reducing work-family interferences and conflicts, compared to Cluster 1 (*in person workers*) and *Cluster 3 (SMEs’ remote workers*); members of the latter cluster believe that technical skills are less necessary to effectively Work from Home compared to members of Cluster 1. Finally, the coping strategy differed among clusters (*F*(4, 158) = 3.22; *p* < 0.05), with *big companies‘ experienced remote workers* showing higher scores on their capacity to positively reinterpret stressful and negative events compared to *lone workers.*

**Figure 1:**
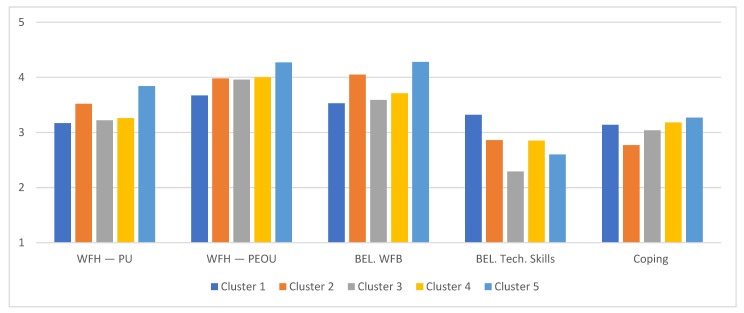
Clusters comparisons on WFH acceptance, remote working attitudes, and coping.

Below, in Figure 1, we graphically summarize the clusters’ average scores on the psychological variables.

## 4. Discussion

This study aims to explore how the adoption of WFH during the second wave of the COVID-19 pandemic influenced and differentiated WFH acceptance, beliefs, and well-being. Specifically, the study tried to identify the work and organizational conditions related to remote workers’ experience and perceptions that lead to the differences across employees. 

The first contribution of this research is the identification of five specific profiles distributed along a continuum from workers with no experience with WFH to experienced workers with a long history of remote work. In addition, the five profiles are related to specific differences in work and organizational conditions. We briefly summarize these differences, and then we discuss the other study implications. 

Cluster 1, called *in person workers*, is composed of workers who perform their job activities in person and within a team. Compared to the other clusters, on average, these workers work closer to their workplace and have a lower education level (equal to a high school diploma or less). The second group, defined as *the lone workers*, is composed of workers who fulfil their job tasks without collaborating with others. The majority of them worked from home during the second lockdown and, compared to the other clusters, they worked remotely fewer days per week. As in Cluster 1, they have a lower education level than the other groups. Cluster 3, the *Small and Medium Enterprises (SMEs)’ remote workers*, comprises employees who Work from Home within a team for Small and Medium-sized companies. On average, they have higher education levels (post-graduate degree or higher). *Big companies’ early-stage remote workers* (Cluster 4) work for large companies in a team, and they started working from home during the first national lockdown. Compared to Cluster 2, these members have a higher educational level. Finally, Cluster 5 is composed of *experienced remote workers* who work in teams in *big companies* and started working from home before the first lockdown. Compared to Cluster 1, they usually spend more time commuting to their physical workplace.

Considering these five profiles and their comparative differences, results show that work and organizational conditions are also related to differences in WFH acceptance, beliefs, and well-being. Specifically, members of Cluster 5 perceived WFH as a simpler, more timesaving, and more productive-effective way to work, compared to other clusters’ perceptions. These results may be explained by the two factors that differentiate this cluster from the others: having previous remote working experience and work in a big-sized organization. These two conditions seem to favor workers’ perceptions of being able to effectively manage WFH by improving the efficacy and effectiveness of their work performance, especially in comparison with those who had never engaged in remote work (*in person workers*) or those who experienced it for the first time during the lockdown (*big companies’ early-stage remote workers*) and/or work in small and medium enterprises (*SMEs’ remote workers*). It is reasonable to assume that employees in Cluster 5 belong to companies that invested resources in designing and implementing remote work before the COVID-19 lockdowns. These companies have probably developed interventions such as training programs, teamwork support, or organizational monitoring and communication, which help create a shared culture of technology use in organizations [26]. Working in this context has probably oriented remote workers’ acceptance of WFH in terms of usefulness and ease of use. In particular, the significant difference in the perception of ease of use of WFH between Cluster 5 and Cluster 1 seems to confirm the Silva Cortès et al. [21] findings that facilitating conditions at the organizational level (such as training, user aids, informational and technical support) improve WFH acceptance and, at the same time, employees’ perception of being capable of managing and controlling the new technological solution. In agreement with preceding literature, this finding suggests that previous direct experience with remote work technologies may decrease employees’ resistance to their use in the workplace [58,68]. Furthermore, members of Cluster 5 reported more positive beliefs about the positive effect of WFH adoption on work-family balance than respondents in Cluster 1 and Cluster 3. There might be two main reasons for these results. Cluster 5 members had experienced remote work before the mandatory WFH, learning over time how to effectively manage home responsibilities and work demands in a feasible balance [57]. Having direct knowledge about WFH benefits for work-life balance probably improved this belief in Cluster 5 members, in comparison with those who had never experienced remote work and usually dedicated less time to commuting (such as Cluster 1 members) or who worked in organizations that, due to limited resources and/or more informal managerial structures, might have a less developed work-family supportive culture and/or less flexible work practices [48], such as Cluster 3 members. The difference between Cluster 5 and Cluster 3 might also be explained by the fact that that the latter group was mainly composed of female workers, whereas Cluster 5 had a higher percentage of males. Women teleworkers are commonly recognized as being more inclined to accept family interference in their work domain compared to men [48]. This tendency, together with a less family-supportive organizational context, might better explain why *SMEs’ remote workers* seem to perceive a more conflictive work-family balance than Cluster 5 members.

Regarding remote work beliefs, *SMEs’ remote workers* report less need for advanced ICT skills to work remotely compared to *in person workers*. This result can be explained by the different education levels of the two groups. Most Cluster 3 members had a master’s degree, which is higher than Cluster 1 members, who reported a high school diploma or lower. Theoretical [20] and empirical [37] studies observed that employees with higher educational levels (primarily academic degrees) are more inclined to adopt remote work technologies. Thanks to their inclination to work based on objectives/results and their openness to non-routine jobs, employees with higher education levels, such as *SMEs’ remote workers*, could have developed the perception that the ICT needed to WFH are less demanding, compared to employees with lower education levels, such as *in person workers.*

Finally, the five clusters highlight workers’ differences in their capacity to cope with the events stemming from the COVID-19 pandemic. The significant difference in coping strategies between Cluster 5 and Cluster 2 suggests the positive influence of team social support in the WFH conditions. According to Kniffin et al. [18], our results seem to confirm that remote workers’ well-being might be positively associated with supportive social interactions with co-workers. In particular, due to their previous remote work experiences and teamwork conditions, members of Cluster 5 may have learned how to promote effective communication and integration with colleagues during the lockdowns. Thanks to this team capacity, co-workers might have been able to ensure sufficient communication to actively participate in team activities (i.e., decision making, task coordination, etc.) and provide a source of social support for teammates in coping with the stressful events of the pandemic [34]. Following Galanti et al. [10], this study also seems to confirm that the social isolation experienced by members of Cluster 2 (*the lone workers*) has probably decreased the opportunity to meet colleagues and, thus, the capacity to effectively cope with the stressful events during the second lockdown.

### 4.1. Strength, Limitations, and Future Directions

One strength of this study is that it provides a picture of employees’ conditions during an understudied phase of the COVID-19 pandemic. Although previous research, have in fact focused especially on the onset of the pandemic, when the novelty, the challenges, and the fear of COVID-19 have for the first time marked employees’ experience, the second lockdown from mid-November 2020 to mid-January 2021, represented a period in which restrictions were reinstated, and gave evidence of great and negative impact on people’s mental well-being (Ben-Ezra et al., 2021; Moradian et al., 2021; Siete et al., 2021).

A second positive point of the study is the detection of worker profiles characterized by work and organizational characteristics, but differing in terms of WFH acceptance, remote work beliefs, and coping strategies. In detecting workers’ profiles, this study grouped respondents into multi-dimensional clusters. These results seem to confirm Ipsen et al.’s [24] suggestion to avoid classifying remote workers using a single characteristic (e.g., gender, age group, or job role) in favor of a more articulated, comprehensive, and situation-driven definition of remote workers. Furthermore, we extended research on technology acceptance, applying the TAM dimensions to a new and the less examined context of the Work from Home during the pandemic.

This study also has several limitations. First, applying snowball sampling through social networks (i.e., LinkedIn, Facebook) might have introduced biases related to researchers’ social network contacts. Future studies should involve a larger audience of respondents possibly unrelated to researchers. Future studies may also extend the data collection to other countries, in order to validate the results of this study. Moreover, we point out that our data collection did not consider several measures that may affect employees’ well-being, such as socioeconomic status (e.g., income status), as well as health conditions. Finally, the study considered a specific period in the evolution of the pandemic and in the experience of remote workers: this is a strength but also a limitation, which affects the generalizability of the results to the whole pandemic. For this reason, future studies should examine, during the pandemic prosecution and in a post-COVID-19 setting, whether the same work and organizational characteristics detect similar or different employees’ clusters and differences in terms of WFH acceptance, motivation, and well-being.

### 4.2. Practical Implications

Despite the exploratory nature of this study, some important practical implications can be outlined. First, based on the profiles that we detected, Human Resource Management Departments might detect and map the five profiles/cluster in their organization’s workforce. Knowing the different levels of workers’ WFH acceptance and orientation might help managers to implement more focused remote work plans and create training actions to support employees’ acceptance. Although employees are interested in training on collaboration tools [69], this can be probably true for employees like the *in person workers* or the *early stage remote workers*, that can benefit of some training about collaboration tools, communication etiquette with colleagues and supervisors, and how to access and navigate the available databases in order to manage their tasks or solve basic IT problems. Second, experienced remote workers (such as Cluster 5 members) could be involved in a different way within the company. For instance, they could be included in teams designing or revising remote work programs. The knowledge gained by these workers from past experience and during the lockdown period might be useful to develop more effective guidelines, protocols, and/or organizational best practices. Third, considering that in our study experienced home workers showed higher coping skills, their active participation in remote work program implementation might help private and public companies to change to more agile and hybrid organizational forms. From this point of view, experienced remote workers might become true change agents [70] capable of helping organizational development through mentoring and/or coaching actions to support less experienced colleagues in accepting and adopting remote work. Moreover, documenting, sharing, and exchanging these experiences with networks of organizations interested in adopting remote work might pool the knowledge about how to foster the necessary organizational learning to build more adaptive and resilient organizations [71]. Finally, according to Pérez-Nebra et al.’s [72] recommendation, institutional actors and HR managers should not forget that the COVID-19 pandemic sharpened a new digital divide between workers who had the possibility and tools to WFH (such as Clusters 4 and 5) and those who did not (such as Clusters 1 and 2). Taking these differences into account is crucial in order to avoid digitalization processes create technological and social gaps among workers.

## 5. Conclusions

The COVID-19 pandemic has required the sudden adoption of mandatory WFH solutions. This exploratory study investigated Italian workers’ experiences during the second stage of the pandemic, between mid-November 2021 and mid-January 2021, and it examined the impacts of the abrupt shift to home work on employees’ WFH acceptance, beliefs, and well-being. Clustering employees based on factors related to telework adoption (i.e., organizational size, remote work previous experiences, work in team, amount of WFH per week, and having a child/children), we found and labelled five different employee groups: (1) *in person workers*, (2) *lone workers*, (3) *SMEs’ remote workers*, (4) *big companies’ early-stage remote workers*, *and* (5) *big companies’ experienced remote workers.* The clusters shared specific homogeneous intra-group member characteristics, and they differ on one or more inter-group member characteristic/s. The five clusters also show significant differences in their members’ WFH experience, remote work beliefs, and coping strategies. Specifically, Cluster 5 members, who work for big companies, work in a team, started working remotely before the national lockdowns, and work this way several days a week, developed higher WFH positive beliefs, greater technology acceptance, and more adaptive coping strategies than other employee groups. Results highlight the need to better comprehend the different WFH experiences and acceptance, and distinguish employees that have to be trained and supported to manage the remote work experience and more experienced employees that can instead be more active and support colleagues and the organization during the remote work program implementation.

## Figures and Tables

**Table 1:** Characteristics of the respondents.

Variables	Total	Variables	Total
Gender, N (%)		Type of employment, N (%)	
Female	67 (41.1%)	Full-time	137 (84.0%)
Male	93 (57.1%)	Part-time	26 (16.0%)
No answer	3 (1.8%)		
Age (years), N (%)		Organizational size, N (%)	
18–24	7 (4.3%)	Micro and Small	43 (26.4%)
25–39	85 (52.1%)	Medium	27 (16.6%)
40–54	56 (34.4%)	Big	93 (57.1%)
From 55	15 (9.2%)		
Have a child/children, N (%)		Work in team, N (%)	
No	90 (55.2%)	No	21 (12.9%)
Yes	73 (44.8%)	Yes	142 (87.1%)
Educational level, N (%)		Job role, N (%)	
Middle school or lower	3 (1.8%)	Top Manager	21 (12.9%)
High school	48 (29.4%)	Middle Manager	13 (8%)
Graduate	25 (15.3%)	Employ	84 (51.5%)
Post-graduate	68 (41.7%)	Technician/Professional	22 (13.5%)
Phd or other Acc. Spec.	19 (11.7%)	Other	23 (14.1%)
Time spent commuting, N (%)		WFH work days, N (%)	
Up to 20 min	69 (42.3%)	0	42 (25.8%)
20- 45 min	61 (37.4%)	1	15 (9.2%)
More than 45 min	33 (20.2%)	2	20 (12.3%)
Remote Work experience, N (%)		3	17 (10.4%)
Never	42 (25.8%)	4	7 (4.3%)
From COVID-19 lockdown	68 (41.7%)	5	58 (35.6%)
Before COVID-19 lockdown	53 (32.5%)	6	4 (2.5%)

Note. N = 163 (in parenthesis, percentage of respondents).

**Table 2:** Means, Standard Deviations and Correlations among study focal variables.

Variable	M	SD	1	2	3	4	5	6	7	8	9	10	11	12
1. Remote Work exp.	-	-	-											
2. Organizational size	2.75	2.11	0.44 **	-										
3. WFH work days	-	-	0.64 **	0.36 **	-									
4. Work in team	-	-	0.11	0.24 **	0.04	-								
5. Having children	-	-	0.19 *	0.15	0.19 *	0.05	-							
6. WFH—PU	3.41	0.84	0.19 *	0.21 **	0.14	−0.08	0.03	-						
7. WFH—PEOU	3.99	0.55	0.02	0.03	0.14	−0.08	0.00	−0.23 **	-					
8. WFH Belief work-family balance	3.83	1.06	−0.16 *	0.03	−0.09	−0.03	0.02	−0.10	0.17 *	-				
9. WFH Belief workplace relationships	3.23	1.17	0.29 **	0.22 **	0.15	−0.05	0.00	0.49 **	−0.32 **	−0.07	-			
10. WFH Belief needed technical skills	2.77	1.25	0.33 *	0.27 **	0.22 **	0.00	0.09	0.33 **	−0.19 *	−0.36 **	0.48 **	-		
11. Coping—Positive reint.	3.12	0.55	0.11	0.09	−0.01	0.24 **	0.04	0.09	−0.07	0.03	0.08	0.07		
12. Work Self-Efficacy	4.22	0.65	0.04	0.06	−0.04	0.11	0.07	0.07	−0.08	0.00	0.14	0.14	0.37 **	-
13. Organizational effect.	3.66	0.66	0.09	0.02	0.12	0.07	−0.13	0.06	0.15	0.03	0.02	0.06	0.18 *	0.03

Note. N = 163. * *p* < 0.05; ** *p* < 0.01.

**Table 3:** Distribution of socio-demographic characteristics across the five clusters (percentages).

Cluster	Gender			Age Groups				Education Level				Employment		Commuting Time		
	F	M	Other	18–24	25–39	40–54	≥55	High School or Lower	Grad.	Master	Ph.D.	Full-time	Part-time	<20 min.	20–45 min.	>45 min.
Cluster 1	47.1	50.0	2.9	8.8	64.7	20.6	5.9	44.1	20.6	29.4	5.9	32.4	67.6	67.6	14.7	17.6
Cluster 2	42.8	57.2	0.0	4.8	52.4	23.8	19.0	52.4	23.8	23.8	0.0	23.8	76.2	33.3	61.9	4.8
Cluster 3	52.9	44.2	2.9	2.9	35.3	55.9	5.9	17.6	11.8	50.0	20.6	11.8	88.2	67.6	26.5	5.9
Cluster 4	41.2	58.8	0.0	2.9	47.1	38.2	11.8	23.5	14.7	44.1	17.6	11.8	88.2	44.1	32.4	23.5
Cluster 5	25.0	72.5	2.5	2.5	60.0	30.0	7.5	27.5	10.0	52.5	10.0	5.0	95.0	27.5	30.0	42.5

Note. N = 163. (F = Female, M = Male).

**Table 4:** Clusters, number of members, and grouping variable scores.

Cluster	N (% of Sample)	Remote Work Experiences	Organizational Size	WFH Work Days	Work in Team	Having Children
		Mean (SD)	Mean (SD)	Mean (SD)	Mean (SD)	Mean (SD)
Cluster 1	34 (20.85%)	1.00 (0.00)	1.82 (0.93)	0.00 (0.00)	2.00 (0.00)	1.26 (0.44)
Cluster 2	21 (12.90%)	1.86 (0.79)	1.76 (0.94)	2.52 (2.33)	1.00 (0.00)	1.38 (0.50)
Cluster 3	34 (20.85%)	2.23 (0.43)	1.61 (0.49)	3.44 (1.52)	2.00 (0.00)	1.56 (0.50)
Cluster 4	34 (20.85%)	2.00 (0.00)	3.00 (0.00)	3.79 (1.47)	2.00 (0.00)	1.44 (0.50)
Cluster 5	40 (24.55%)	3.00 (0.00)	3.00 (0.00)	3.73 (1.72)	2.00 (0.00)	1.55 (0.50)
TOTAL	163 (100%)	2.07 (0.76)	2.31 (0.86)	2.75 (2.11)	1.87 (0.34)	1.45 (0.50)
*F*		159.72 **	47.50 **	37.00 *	^a^	2.14
dF (Within; Between)		(4; 158)	(4; 158)	(4; 158)	-	(4; 158)

Note. N = 163. * *p* < 0.05; ** *p* < 0.01. ^a^ Impossible to compute because there was no within-group variance. Each group is homogeneous on this characteristic.

**Table 5:** Clusters and respondents’ characteristics variables.

Cluster	Gender	Age Groups	Educat. Level	Employment	Commuting
	Mean (SD)	Mean (SD)	Mean (SD)	Mean (SD)	Mean (SD)
Cluster 1	1.52 (0.51)	2.24 (0.70)	1.97 (1.00)	1.68 (0.47)	1.41 (0.50)
Cluster 2	1.57 (0.51)	2.57 (0.87)	1.71 (0.84)	1.76 (0.44)	1.71 (0.56)
Cluster 3	1.45 (0.51)	2.65 (0.65)	2.74 (0.99)	1.88 (0.33)	1.74 (0.79)
Cluster 4	1.59 (0.50)	2.59 (0.74)	2.56 (1.05)	1.88 (0.33)	1.79 (0.81)
Cluster 5	1.74 (0.44)	2.43 (0.67)	2.45 (1.01)	1.95 (0.22)	2.15 (0.83)
TOTAL	1.58 (0.49)	2.48 (0.72)	2.34 (1.04)	1.84 (0.34)	1.78 (0.76)
*F*	1.77	1.79	5.13 **	3.21 *	4.83 **
dF (Within; Between)	(4; 155)	(4; 158)	(4; 158)	(4; 158)	(4; 158)

Note. N = 163, * *p* < 0.05; ** *p* < 0.01.

**Table 6:** Clusters, WFH acceptance, remote work attitudes, coping strategy, work self-efficacy and organizational effectiveness.

Cluster	WFH—PU	WFH—PEOU	Belief W-F Bal.	Belief Work. Relat.	Belief Tech. Skills	Coping	Work Self-Eff.	Organ. Eff.
	Mean (SD)	Mean (SD)	Mean (SD)	Mean (SD)	Mean (SD)	Mean (SD)	Mean (SD)	Mean (SD)
Cluster 1	3.17 (0.76)	3.67 (0.52)	3.53 (1.05)	3.18 (1.14)	3.32 (1.22)	3.14 (0.53)	4.25 (0.50)	3.60 (0.77)
Cluster 2	3.52 (0.63)	3.98 (0.44)	4.05 (1.02)	2.48 (0.98)	2.86 (1.01)	2.77 (0.49)	4.03 (0.76)	3.54 (0.68)
Cluster 3	3.22 (1.00)	3.96 (0.49)	3.59 (1.18)	3.03 (1.16)	2.29 (1.17)	3.04 (0.53)	4.26 (0.56)	3.66 (0.63)
Cluster 4	3.26 (0.88)	4.00 (0.52)	3.71 (0.97)	3.50 (1.31)	2.85 (1.21)	3.18 (0.48)	4.39 (0.59)	3.70 (0.55)
Cluster 5	3.84 (0.66)	4.27 (0.55)	4.28 (0.93)	3.08 (1.16)	2.60 (1.33)	3.27 (0.62)	4.21 (0.78)	3.73 (0.55)
TOTAL	3.41 (0.84)	3.99 (0.54)	3.83 (1.06)	3.23 (1.17)	2.77 (1.25)	3.11 (0.55)	4.22 (0.65)	3.66 (0.66)
*F*	4.55 **	6.29 **	3.39 *	1.27	3.33 *	3.22 *	1.29	3.55
dF (Within; Between)	(4;158)	(4; 158)	(4; 158)	(4; 158)	(4; 158)	(4; 158)	(4; 158)	(4; 158)

Note. N = 163. * *p* < 0.05; ** *p* < 0.01.

## Data Availability

The data presented in this study is not publicly available due to ongoing analysis but are available from the corresponding author on reasonable request.
